# Automated overview of complete endoscopies with unsupervised learned descriptors

**DOI:** 10.1007/s11548-025-03502-1

**Published:** 2025-08-27

**Authors:** O. Leon Barbed, Pablo Azagra, Juan Plo, Ana C. Murillo

**Affiliations:** DIIS-i3A, University of Zaragoza, Zaragoza, Spain

**Keywords:** Endoscopy, Representation learning, Scene classification, Unsupervised learning, Video summarization

## Abstract

**Purpose:**

We aim to automate the initial analysis of complete endoscopy videos, identifying the sparse relevant content. This facilitates long procedure recording understanding, reduces the clinicians’ review time, and facilitates downstream tasks such as video summarization, event detection, and 3D reconstruction.

**Methods:**

Our approach extracts endoscopic video frame representations with a learned embedding model. These descriptors are clustered to find visual patterns in the procedure, identifying key scene types (surgery, clear visibility frames, etc.) and enabling segmentation into informative and non-informative video parts.

**Results:**

Evaluation on complete colonoscopy videos presents good performance identifying surgery segments and different visibility conditions. The method produces structured overviews that separate useful segments from irrelevant ones. We illustrate its suitability and benefits as preprocessing for other downstream tasks, such as 3D reconstruction or video summarization.

**Conclusion:**

Our approach enables automated endoscopy overview generation, helping the clinicians focus on the relevant video content such as good visibility sections and surgery actions. The presented work facilitates faster recording reviewing for clinicians and effective video preprocessing for downstream tasks.

**Supplementary Information:**

The online version contains supplementary material available at 10.1007/s11548-025-03502-1.

## Introduction

Endoscopies are a more and more frequent component of health screening programs in numerous countries to prevent late, and possibly more severe, diagnoses. These preventive programs, together with the common use of the procedure to monitor existing patients, produces lots of interventions. In endoscopy procedures, a trained physician manipulates an endoscope to navigate through hollow organs of the patient. The endoscope is a flexible and slim tube which has precise controls on the practitioner’s end and medical tools on the other end, including a camera, to perform the exploration.

In this work, we focus on colonoscopy, one of the main procedures for gastro-intestinal (GI) tract exploration. During this very frequent medical practice, large amount of footage in the form of endoscopic video recordings is generated. Recent large image and video endoscopy datasets like HyperKvasir [1] and EndoMapper [2] show the medical community’s interest on research to improve the treatments in the field. These datasets are also directed toward automation and assistive methods [3] that can speed up and even offer new possibilities for the treatment.

Applications of AI in GI endoscopy data have found promising results on 3D modeling [4], polyp detection [5], or hemorrhage segmentation [6]. However, direct processing of complete endoscopy videos is often unrewarding because of the sheer volume of data and the frequent lack of visibility (caused for example by occlusions or by the endoscope hitting the bowel’s walls). The navigation is complicated and usually the camera visibility is blocked by walls and liquids rendering large chunks of the video non-informative. Colonoscopy recordings, which can be more than 30min long, typically imply very costly manual process to achieve a thorough review. Procedures to automatically split the main parts of these recordings can save experts time without decreasing quality, and could be directly integrated in the standard workflows.

This work presents a novel approach to automatically obtain a semantic overview of the complete endoscopic video. The main steps are: (1) Well-known *representation learning* methods to obtain specific descriptors for endoscopic images. (2) Discovery of the *semantic classes* with a human in the loop. Clustering the learned descriptors identifies relevant scene types in this domain. A human assigns semantic labels once to the clusters obtained, producing the final set of semantic classes. (3) *Semantic video partitioning* with different classification heads using these semantic classes.

The main contributions in this work are the presented framework for automated overview generation^1^ and the demonstration in complete colonoscopy recordings. Our experiments on a public dataset illustrate the informative overviews obtained for real colonoscopies, identifying key parts (surgical actions) and the different visibility conditions along the recording. The obtained overviews can (1) save practitioners time when reviewing the recordings and (2) are shown to facilitate the execution of other automated tools, by running them only on relevant parts of the recording.

## Related work

*Image representation learning.* There is great interest in learning models that capture representative features of the training data in order to use it later for different downstream tasks. From the different approaches in self-supervised methods, *contrastive learning* has obtained great results. Instead of defining an pretext task to supervise the description (as [7] demonstrated in endoscopy), the supervision affects the distribution of the descriptors in the latent space. In contrastive learning, the descriptor of an input is considered good if it is close in the latent space to other inputs that come from similar sources (positive samples) and far away from inputs of different sources (negative samples). Many recent approaches, such as MoCo [8] or SimCLR [9] are based on contrastive learning, attempting to map semantic differences between data points into their learned representations. BYOL [10] is similar in that it trains an online network to match the output of a parallel target network for a different augmented view of the same source image. It adds another prediction module with a learnable transformation on the online network to create asymmetry. The contrastive learning is performed only with positive samples, which makes the training more robust and efficient. It presents a flexible and easy to implement framework, with lower requirements than other approaches by using only positive samples. Note that our approach is also compatible with other very similar alternatives to BYOL such as SimSiam [11], or a foundation model for endoscopy such as EndoFM [12].

*Medical video summarization.* Many medical procedures are lengthy and result in hours of recorded footage. Consequently, and similar to our goal, several works in the literature attempt to summarize these recordings by identifying the relevant parts or segments in the video. For example, Byrnes et al. [13] propose a framework that analyzes motion and description of the frames to create a segmentation of bronchoscopic videos and compute coverage maps. Meyer et al [14] proposed a method that uses on-the-fly annotation in live video to improve the result and reduce the amount of reviewing. Wang et al. [15] proposed a convolutional LSTM network to extract non-informative frames from surgical nasal endoscopic videos with a previous step to segment the outside frames. Many approaches for medical video summarization use selected descriptors with unsupervised learning, such as clustering or distance selection. For instance, Raut et al. [16] use a learned descriptor to cluster and discard redundant frames from the long videos and reduce the amount of time needed to review the procedure. Using classic descriptors, Maher Ben Ismail et al. [17] discard the noisy frames of the video with an unsupervised clustering method that determines the number of clusters from a maximum initial number. Mehmood et al. [18] also use classic descriptors to select a keyframe based on significant differences with the previous keyframe. They summarize the videos using these keyframes to reduce the bandwidth usage for wireless shipment of the recording. Similar to these works, our approach serves as preprocessing to facilitate further analysis of this data by medical staff or with automated tasks, such as 3D reconstruction algorithms. However, instead of only choosing keyframes as a summary or discarding noisy frames, we propose a method that segments the whole video into different clips with an associated semantic label. Our approach does not assume predefined classes, but follows self-supervision to find representative types of scenes during the setup of the system.

*Colonoscopy video segmentation.* Existing approaches for colonoscopy video analysis are mostly focused on the frame-level analysis. van der Putten et al. [19] present an approach to classify the images of the video into dysplasia (abnormal cells) and non-dysplasia categories. Similarly to them, Pacal et al. [5] also obtain the bounding box in the frames where polyps are found. More similar to our goals, Ali et al. [20] develop an approach that analyzes the frames of the video looking for artifacts (specularity, bubbles, blur, etc.) to segment a corresponding bounding box. This bounding box could be used to ignore those zones of the images on following steps. A natural improvement explored in other works and in ours is to incorporate temporal cohesion to the frame-level analysis. Byrne et al. [21] present a work that evaluates each frame into polyp categories and then uses a credibility update mechanism that gives a score between 0% to 100% taking into account the info from previous frames. Other works, like Yu et al. [22], consider short video clips as 3D blocks and train a 3DCNN model with them. More closely aligned with our work, Liu et al. [23] uses three different flows to segment non-informative frames from small colonoscopy sequences. However, these methods fail to capture the information of a video in large temporal windows. To prevent the large temporal window information loss, works like Boers et al. [24] use a 2D network as a feature extractor and learn the temporal information through a RNN. Harada et al. [25] present an approach that uses clustering to segment the video and to improve the temporal stability. Our framework is focused on self-supervision to obtain segmentation, similar to works outside of medicine such as [26] and [27] but adding minimal supervision once to obtain concrete and constant semantic classes. We do not segment into given label classes, but into semantic classes that emerge when analyzing complete recordings from real colonoscopies. A discussion on similar relevant methods is included in Online Resource 1.

**Fig. 1:**
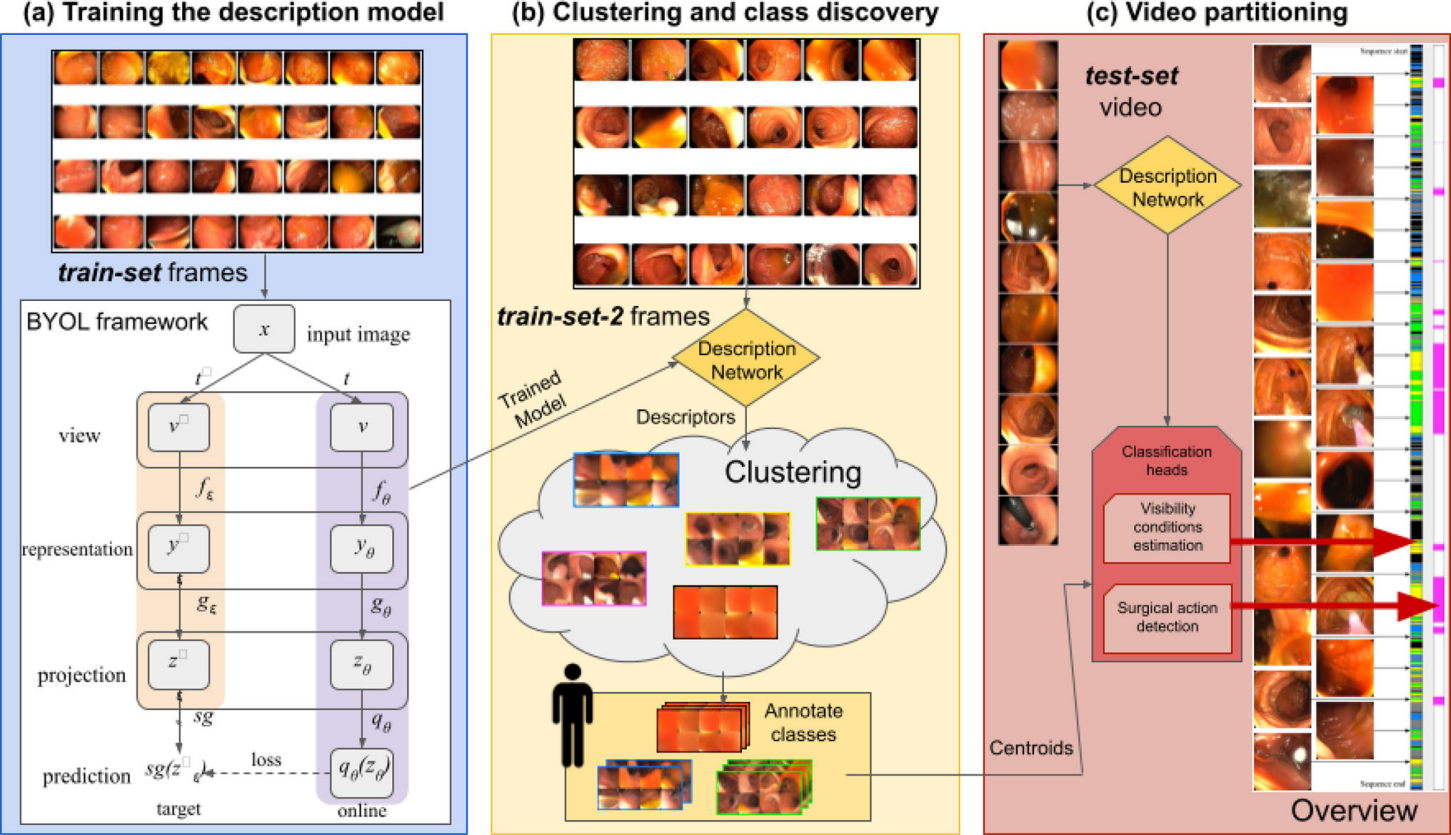
Overview of the proposed framework. **a** the first step uses BYOL to learn a description model that provides good image representation (descriptors). **b** the second step uses that model to describe endoscopy video frames and cluster these descriptors to identify scene types (classes) along these videos. A human inspects the emergent classes to assign semantic labels to them. **c** the third step segments the complete video into clips corresponding to the discovered classes

## Automatic overview of endoscopy recordings

The proposed automatic overview approach takes as input a video recording of and endoscopy procedure and divides it into semantically consistent segments. Different classification heads label these segments to highlight relevant information in the final overview. Our method is summarized in Fig. 1, and detailed in the next subsections.

### Image description and class discovery

We choose the *BYOL* [10] *contrastive learning* framework to obtain representative descriptors of the endoscopy frames (Fig. 1a). BYOL is used to train an image description model without the need for supervision or negative samples, creating a domain-specific description network with low resource consumption. The trained description model infers descriptors for a second training set of frames. Our goal is to *discover the scene classes* that are prevalent within our domain from these descriptors (Fig. 1b). In order to achieve this, we cluster the extracted descriptors using *K*-means. To limit the computational budget and annotation time, we choose the smallest number of clusters that separates the frames into clearly defined classes, $k=100$, and set a maximum number of iterations of 300. Online Resource 1 includes an analysis on the explored values of *k*. We organize the resulting clusters through visual inspection into a hierarchical class structure with two levels. The first level in the class hierarchy separates frames that are *informative* (the visibility conditions are adequate to identify different elements like tissue, haustra, etc.) to from those that are *non-informative*. The second level breaks down the previous classes into more specific semantic classes. For instance, our descriptors are able to segregate frames with surgical tools present into an informative class called *Surgery*. A detailed list of the semantic labels, as well as examples, is provided in Sect. 4. Once the clusters and their labels are defined, the centroid of each cluster is stored as part of the model.

### Semantic video partitioning

Our proposed approach to obtain an *automatic overview* firstly obtains the descriptors for all the frames in a test video using our description model. Then, we compute the smallest distance of each frame to each labeled class ${d_{L} =\min \limits _{n=1\dots N_L}\left( \Vert x-c^{n}_L \Vert ^{2}_{2} \right) ,}$ where $d_{L}$ is the distance from the frame $f_i$ to class *L*, $N_L$ is the number of centroids that correspond to class *L*, *x* is the descriptor of the frame $f_i$ and $c^{n}_L$ is the *n*-th centroid of class *L*. After this, the distances go to different classification heads that detect relevant information to the final overview. In our experiments, we consider two tasks: visibility conditions estimation and surgical action detection.

The *visibility conditions estimation head* labels each video frame depending on its distance to each class. We apply a robustness filter to assign the frame label:

1 $$\begin{aligned} { \text{ class }(f_i)= {\left\{ \begin{array}{ll} L,&  \text {if } d_1<d_2 * R\\ \text {Uncertain}, &  \text {otherwise} \end{array}\right. } } \end{aligned}$$

where $d_1$ is the minimum distance to the closest class and $d_2$ is the minimum distance to the second closest class. *L* is the label assigned to $f_i$. *R* is a threshold ($0<R\le 1$) used to set unclear (borderline) cases. A new class (*Uncertain*) is created to deal with these borderline cases. To obtain a more robust segmentation, we perform a *temporal consistency check* of these labels to obtain smoother segments. The label of the $i^{th}$ frame is set as the *mode* of all the labels within a window of *M* frames, centered in the $i^{th}$ frame. Short segments are labeled as *Uncertain* if they contain less than *P* frames.

For the *surgical action detection head*, only the distances to the *Surgery* class $d_{\text {Surgery}}$ are used. We smooth the result using a moving average with window size *W* and threshold *t* it so frames with $d_{\text {Surgery}}<t$ are labeled as *Surgery*.

While we focused on these two problems, our description system is generic enough to allow more classification heads to extend the overview content. In Online Resource 1, we consider an alternative to our classification heads: a neural network trained on the labeled clusters. In our test, this approach achieves only slightly lower accuracy, but its simpler architecture could be more robust and generalizable in other domains.

## Experiments

Our experiments are run on the EndoMapper (EM) [2] dataset. It contains complete endoscopic procedures of the gastro-intestinal (GI) track recorded during daily medical practice. We chose 61 colonoscopy sequences randomly for different stages of our experimentation. The training sets are composed of one in ten frames from the complete videos. All frames are center-cropped into a squared shape, and the resolution is reduced to 224$\times$224. The sequences are organized into the following subsets: *train-set* (35 videos, 162739 frames. *train-set-2* (20 videos, 233015 frames), *test-set* (6 complete videos). Details on the exact videos used are in the Online Resource 1.

### Class discovery and classification setup

We use the BYOL framework and the *train-set* to train a ResNet50 network (pre-trained on ImageNet) which runs at 200fps. More training details in the Online Resource 1. The output of the model, a vector of size 2048, is used as frame descriptor.

**Fig. 2:**
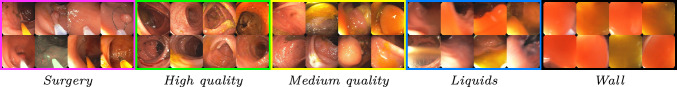
Sample images of clusters for the five fine-grained classes identified by our system

We apply the learned *description model* on the *train-set-2*, and cluster all the obtained descriptors via *K*-means ($k=100$) to identify possible scene types in the data. Visual inspection shows that a dominant semantic class emerges for a significant number of the obtained clusters, as it can be seen in the examples from Fig. 2. This points to good representativeness of the learned descriptor in this domain. The *obtained clusters* were manually inspected by two different annotators. Both annotators assigned the same semantic class to 91$\%$ clusters. The annotators agreed on the labels of the remaining clusters after a brief discussion. The semantic classes considered are organized hierarchically into a first binary level (informative vs. non-informative) and a second level with fine-grained labels.

*Informative classes.* Frames with potential relevant information for medical staff, artificial intelligence algorithms or both. $\textit{66}$ out of the 100 *clusters* belong to this class. It is split into the following fine-grained classes:

- *Surgery:* 6 clusters. A surgical tool of any kind is visible. Specially useful in diagnostic endoscopies: they contain the most information about the procedure.
- *High quality:* 35 clusters. Frames with clear organ view and few artifacts (e.g., motion blur, liquids). These segments record where the practitioner was able to inspect the organ, and they are also good candidates for 3D reconstruction pipelines.
- *Medium quality:* 25 clusters. Frames where the organ is visible to some extent, but there is a presence of artifacts that hinder the visibility. They may be informative for medical use but not for 3D reconstruction pipelines because of their noisy content.

*Non-informative classes.* Frames that are noisy or contain no information. These frames are not relevant for medical staff nor algorithms. 34 out of the 100 *clusters* belong to this class. It is split into the following fine-grained classes:

- *Liquids:* 21 clusters. Frames where there are liquids blocking the view, from the water pump or otherwise. They are not typically useful for exploration or 3D reconstruction, but they can help us to detect and/or time the cleaning operations.
- *Wall:* 13 clusters. The endoscope camera is up against the organ wall. They contain no visual information, so they can be discarded in later post-processing of the video.

In addition to these classes, in our video segmentation experiments, we assign the *Uncertain* class to frames that do not get classified with enough confidence (we set $R=0.95$). Note that *Uncertain* sections are important in the final overview since they could contain information hard to classify correctly, so they are risky to discard. A final *temporal consistency* check is run with a sliding window of $M=40$ frames and a filter of segments shorter than $P=80$ frames (Online Resource 1, Parameter tuning). This step outputs the final semantic video partitioning of the system.

### Surgical action detection and visibility estimation

**Fig. 3:**
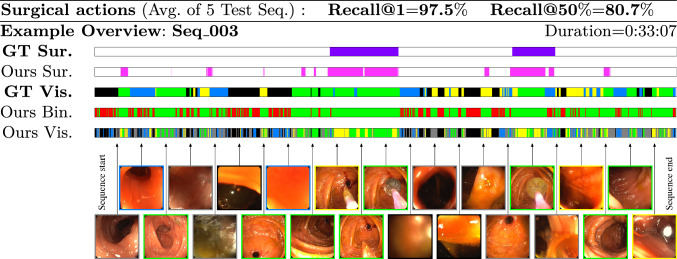
Surgical action detection results and example of automatic video overview obtained with our approach. Horizontal bars represent the video from start to end (left to right). The different colored sections are: annotated (purple) and predicted (pink) surgical actions. Binary predictions for *informative* (green) and *non-informative* (red). Fine-grained predictions: *high quality* (green), *medium quality* (yellow), *liquids* (blue) and *wall* (black). Bottom images are frame samples

This experiment evaluates the automatic overview obtained by our approach, both the *surgical tool* (Sur.) and *visibility condition* (Vis.) results are summarized in Fig. 3.

The *surgical actions* head is evaluated on six sequences with the frames containing surgical tools manually annotated (*GT Sur.*). The most critical aspect for the resulting overview is that all surgery intervals are found. Then, we report the recall in our predictions (*Ours Sur.*) at two levels: at least one frame is detected (Recall@1), and at least half of the frames of the surgery interval are detected (Recall@50%). Sequence *Seq_036* from *train-set-2* is used to adjust the parameters. We establish the window size of the moving average window size to *W*=400 frames, and the threshold to *t*=6.3 to maximize Recall@50% for this labeled sequence. We tested the performance on the remaining five labeled sequences (*test-set* except Seq_011, that does not have surgery) obtaining *97*.*5*% average Recall@1 (our system only missed one surgical action in total, error analysis of this action included in Online Resource 1) and *80*.*7*% average Recall@50%, which means that our system also covers most of the actions’ spans.

The *visibility conditions* head is evaluated on the class hierarchy explained in Sect. 4.1. Note each *Surgery* cluster is re-labeled as the most relevant visibility class to have a dense annotation, because *Surgery* class is not related to visibility conditions. We manually labeled *Seq_003* with the four fine-grained visibility classes (*GT Vis.*). Each segment in our predictions, *Ours Vis.*, is considered correct if the predicted label corresponds to the *mode* of *GT Vis.* in the corresponding interval. To obtain the binary informative vs non-informative segmentation (*Ours Bin.*), we do the same but using the corresponding super-classes in *GT Vis.*. The results of the classification in the binary and fine-grained visibility condition classes are summarized in Table 1. The binary segmentation is the most critical for downstream tasks. Note the high *93*% *precision* and *88*% *recall* for *Informative* segments, and *92*% and *87*% for *Non-Informative*, respectively. The fine-grained segmentation presents lower recall (around 60% for all classes except Medium quality, which has the more heterogeneous content, but the most frequent confusion is within Informative labels, therefore not that critical for potential uses in downstream tasks). Notably, our system does not rely on predefined labels, but includes the discovery of semantic classes in the data without prior knowledge or assumptions. Figure 3 shows a qualitative example of the results in Seq_003. The remaining five *test-set* sequences do not have visibility labels, but we include *qualitative results* of the automatic overview generated by our system on them in Online Resource 1.

To illustrate *potential generalization* of the approach, Online Resource 1 also includes several overviews obtained for a different type of endoscopy (gastroscopy). The results look promising, given the domain shift, although adaptation to the new domain (with updated clustering and labeling) would be necessary for its application in downstream tasks. Different downstream applications require human labeling of the clusters (types of scenes) that represent the relevant semantic information for the task. This is a current limitation for the versatility of the system. However, note this labeling happens at cluster-level, which is much less time-consuming than frame-level.

**Table 1:** Results on visibility assessment for *Seq_003*. (Top) Confusion matrix for informative versus non-informative segmentation. (Bottom) Confusion matrix for segmentation with four fine-grained labels

	Predicted label			
True label	Info.	Non-Info.		
Info.	${\textbf {113}}$	16		
Non-Info.	8	${\textbf {104}}$		
Info. *P* = **93**% *R* = **88**%				
Non-Info. *P* = **92**% *R* = **87**%				

	Predicted label			
True label	High	Medium	Liquids	Wall
High	${\textbf {41}}$	28	6	2
Medium	21	${\textbf {23}}$	8	0
Liquids	1	5	${\textbf {33}}$	17
Wall	0	2	16	${\textbf {38}}$

Bold values used to highlight the highest value of each class, i.e., highest value of each row

*Reviewing efficiency*. Another benefit of our automated overview is the quick analysis of non-relevant video content. We observe that nearly all analyzed sequences contain more than $30\%$ of their video frames identified as *Non-Informative* (Avg. $35\%$), which is nearly 10 min of real time per sequence. Our automatic overviews can bring a significant boost in reviewing efficiency and help medical staff to review videos in less time, or allow automated algorithms to process directly segments with potentially relevant information, as illustrated in the following experiment.

### Preprocessing for downstream tasks

**Fig. 4:**
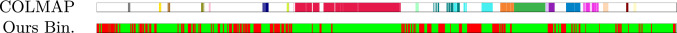
COLMAP reconstructions compared to our binary classification on Seq_003

Additionally, our proposed approach assists other tools for downstream tasks, for example 3D reconstruction. In particular, we run the structure-from-motion pipeline COLMAP. Figure 4 illustrates how COLMAP, assisted by a feature extractor specific for endoscopy [28], obtains 3D reconstructions on several regions of *Seq_003* after processing the complete video. Notably, the reconstructions happen almost exclusively in sections our approach labels as *Informative*; therefore, processing only these segments would obtain the same results while avoiding more than a third of the workload.

## Conclusions

This work proposes an approach for automatic overview generation of complete endoscopy recordings. Our approach includes a semantic class discovery step, where frequent semantic categories emerge from the clustering of video frames using the representation learned. After defining the target semantic classes, our approach considers different classification heads that recognize relevant and complementary information (surgical actions and visibility conditions) to compose the final overview. The system requires minimal human supervision during the class definition, and is completely automatic afterward. Our validation is run on complete colonoscopy videos acquired during real medical practice and manually labeled. The overviews obtained facilitate automatic processing and exploration of large and noisy real recordings, by reliably separating non-informative segments from interesting parts of the recording, such as parts where surgical tools are visible or with good visibility of the organ. This could save close to 10 min per video (around 1/3 of the total duration) of expert practitioners during the video review, and can also automatically identify relevant segments to be processed by algorithms targeting different downstream tasks such as more accurate semantic analysis or 3D reconstruction. Future research steps could consider additional semantic classes to expand the downstream applicability of the method.

## Supplementary Information

Below is the link to the electronic supplementary material.

Supplementary file 1 (pdf 20790 KB)

## Footnotes

1 Models and code are being released to the community upon acceptance.
