# Supplementary material for: Automated overview of complete endoscopies with unsupervised learned descriptors
Source: Int J Comput Assist Radiol Surg. 2025 Aug 27;21(3):617–24. doi: 10.1007/s11548-025-03502-1 (PMC13035558; doi:10.1007/s11548-025-03502-1)
Supplement: Supplementary file 1 — (pdf 20790 KB) [file 11548_2025_3502_MOESM1_ESM.pdf]

Automated overview of complete endoscopies with unsupervised learned descriptors

Online Resource 1

O. Leon Barbed[1*], Pablo Azagra[1], Juan Plo[1], Ana C. Murillo[1]

[1]DIIS-i3A, University of Zaragoza, Zaragoza, Spain.

*Corresponding author(s). E-mail(s): leon@unizar.es;

1 Experimental setup

The data from the EndoMapper [1] dataset used is:

- **train-set**: 35 videos (Seq_001, 002, 004, 005, 006, 007, 008, 009, 012, 013, 017, 024, 030, 037, 039, 041, 074, 052, 053, 060, 063, 072, 076, 078, 081, 084, 089, 090, 091, 092, 095, 096), for a total of 162739 frames.
- **train-set-2**: 20 videos (Seq_010, 014, 015, 018, 019, 020, 025, 026, 031, 035, 036, 052, 044, 048, 058, 061, 067, 074, 079, 086), for a total of 233015 frames.
- **test-set** of 6 complete videos (Seq_003, 011, 016, 022, 093, 094). Additional gastroscopy test sequence (Seq_021).

The configuration to train the description model with the BYOL framework is: The network's MLP hidden size is 4096, and the projection head, 256. The optimizer used is an SGD with learning rate of 0.01, momentum of 0.9 and weight decay of 0.0004. The batch size is 64 and the momentum update is 0.99. We performed data augmentation of random horizontal flips, random shifts in brightness (±0.8), contrast (±0.8), saturation (±0.8) and hue (±0.2) and 20% probability of changing to grayscale. The network was trained for 10 epochs, but the model checkpoint with the lowest loss value was obtained after the 7th epoch, so those are the model weights used in the experiments.

2 Additional experiments

Video segmentation with semantic information

We also show how using our video segments to guide **video summarization** pipelines produces more varied and complete summaries of complete endoscopies. Figure 1 shows how current general-purpose approaches for video summarization risk information loss by skipping large chunks of the video. This risk is too high for healthcare applications, so we propose using video segmentation techniques to guide the summarization. We run the recent video summarization approach UBiSS [2], which outputs a saliency score for each frame. We set a 200-frame budget for the summaries in this experiment. Blindly selecting the highest saliency frames as suggested by the method results in large video chucks being absent in the summary. To mitigate this risk, we incorporate video segmentation as guidance so the summary contains at least one frame per video segment. This can be performed via popular video segmentation tools such as FFmpeg [3], but it lacks semantic information to set domain-specific priorities between segments. The semantic segmentation performed by our approach lets us set a "class priority" for the different segments: we only include one representative frame from the *Non-Informative* segments, while we keep including *Informative* frames until the summary reaches the budget limit. The result is a more complete summary that gives a more complete overview of the procedure. It preserves the representation of all the separate segments of the video, *Informative* or not, while giving more representation to the former since they are the most relevant.

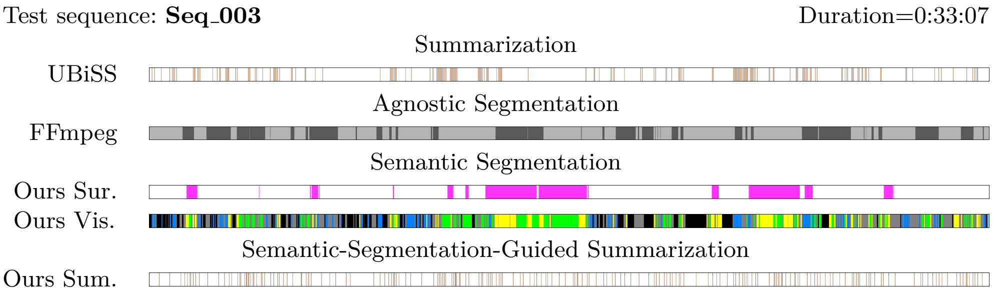

Fig. 1 Video summarization example. UBiSS and Ours Sum. are both summaries, where brown lines indicate the frames included in the summary. FFmpeg bar represents in alternating shades of gray the different segments without semantic classification. Ours Sur./Vis. are the outputs from our segmentation system.

CNN classifier alternative to our classification heads

Our system classifies test frames following a Nearest Neighbor algorithm using the learned descriptors and the labeled clusters. In this experiment we compare the performance of a different classification method: training a neural network using the labels of the clusters as supervision. We use the already trained ResNet50 model for image description and add an MLP at the end to learn the final classification. Table 1

contains the results of some of the best variations obtained using this method. Training directly using all the clustering data with the associated cluster labels did not perform well (top rows obtain less than 48% F1-score). To refine this process, we filtered the images by their distance to the centroids they were assigned to in the clustering. Additionally, for the Surgery class, we manually selected the images that contain surgical tools. Note that this per-image labeling is much more intensive than our proposed per-cluster labeling, hence why we only applied it to the Surgery class. Still, the **best performing model**, which counted with two extra hidden layers with 1024 and 256 embedding size, obtained a per-frame F1-score of **52.0**% across the 5 classes. Our model, when for a fair comparison only used one classification head for the five classes instead of two (treating Surgery the same as it was one of the visibility classes), obtained **52.9**%. This result certifies that statistically our proposed approach is more accurate, albeit not by much. Figure 2 shows the visual output of both methods. Visually, the results are also very similar. Thanks to the manual per-frame labeling performed to train the MLP version, it is able to better segment the Surgery sections (pink). Even though when taking into account all classes, the proposed Nearest Neighbor approach obtains better metrics, the MLP alternative has certain benefits in finding Surgery segments, suggesting favorable use cases when strong supervision is available.

Table 1 Performance of several variations of MLP classification heads added to our description network to perform direct classification of frames. Different MLP architectures and training subsets are used to fully compare the performance against the current method (bottom row).

**Model**	**Classifier arch.**	**Classifier training data**	**F1-score**
BYOL+MLP	MLP: 1024, 256, 64	All training data with manual cluster labels	47.1
BYOL+MLP	MLP: 1024, 256	All training data with manual cluster labels	47.2
BYOL+MLP	MLP: 1024, 256	Surgery class: manual image labels — Other classes: Closest to the class centroid (all images)	50.8
BYOL+MLP	MLP: 1024, 256	Surgery class: manual image labels — Other classes: Closest to the class centroid (<75th percentile)	50.1
BYOL+MLP	MLP: 1024, 256	Surgery class: manual image labels — Other classes: Closest to the class centroid (<50th percentile)	52.0
BYOL+MLP	MLP: 1024, 256	Surgery class: manual image labels — Other classes: Closest to the class centroid (<25th percentile)	49.1
**BYOL+NN**	-	Cluster manual labels	**52.9**

Parameter tuning

Our method is configured to obtain the best performance in real endoscopy videos. We performed experiments to discover the reliance of the system on each parameter,

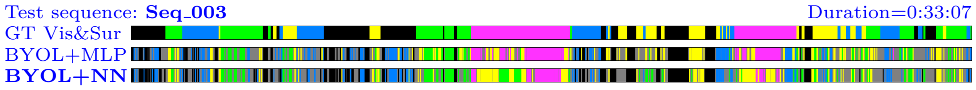

Fig. 2 Video segmentation comparison between our proposed system (BYOL+NN) and an alternative with a neural network as its classifier (BYOL+MLP).

finding the optimal values for this domain. We explore the values for the parameters R, M and P in Figure 3. The left graph shows how as the ratio between the two closest distances approaches 1, the ratio between incorrect and correct classifications increases. This fact justifies setting a ratio threshold (R) to soften as many of the bad classifications as possible while not degrading the predictive power of the system. The green dashed line represents the value chosen ($R = 0.95$), meaning that our system classifies as *Uncertain* all the frames to the right of it, which are overwhelmingly incorrect. The top-right graph shows the effect of different values of M in our system, which regulates the size of the smoothing performed on the full sequence predictions. The local maximum found for F1-score at the value $M = 40$ justifies this as the optimal value. Bottom-right graph shows the effect of varying the P parameter, which changes the predicted class of small segments to *Uncertain*. Smaller segments are harder to predict correctly and usually avoiding classifying them tends to always improve the metrics, i.e. F1-score always increases with P. However, this means a larger portion of the sequence being *Uncertain* in the predictions, reducing the utility of the system. The decision made was to choose the highest value of P before the F1-score slows its growth, i.e. $P = 80$.

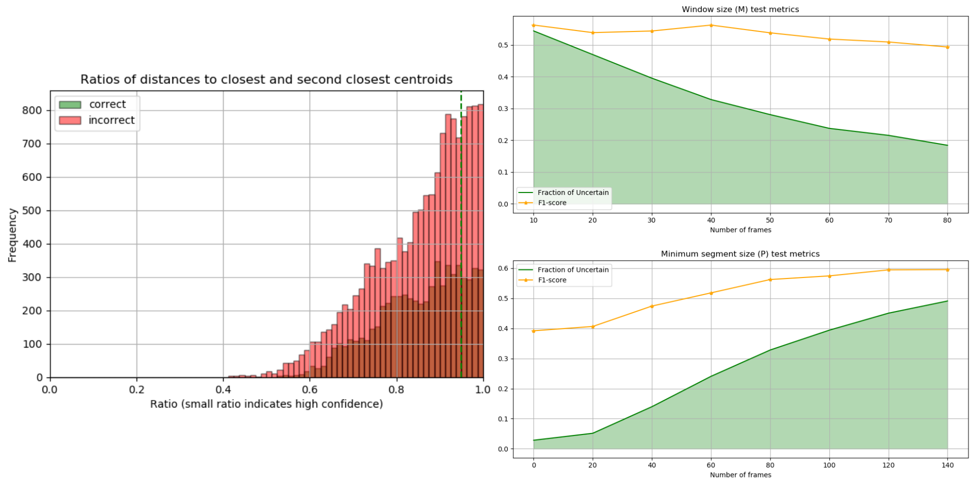

Fig. 3 Ablation study of the model's parameters for optimal tuning. Left: Histogram of the ratio between the nearest cluster and the second closest to test images that are between an *Informative* and a *Non-Informative* class cluster. Correct images are the ones where the closest cluster is the true label. The vertical green dashed line represents the value chosen for $R = 0.95$. Right: F1-scores and fraction of frames classified as *Uncertain* for different values of the parameters M (top) and P (bottom).

We also study the possibility of using smaller numbers of clusters in the K-means algorithm, because it requires less computation to process. However, as we observe in Figure 4, with $k = 50$ the Surgery frames are spread over multiple clusters next to other non-Surgery frames. This poses a problem in manual labeling of the clusters and later classification that does not allow our system to identify Surgery frames. For this reason, the value chosen for k is 100, where there are Surgery-only clusters, adding classification power to the whole system. Using $k = 100$, the clustering reaches a Silhouette score of 0.08 and a Davies-Bouldin Index of 2.29.

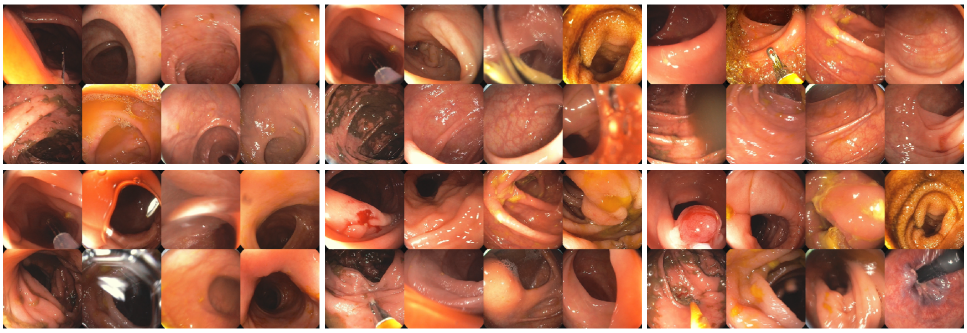

Fig. 4 Cluster examples with a smaller value for k in the K-means algorithm: $k = 50$.

Image description: embedding-space visualization

For the image description network, there are a few unsupervised methods that can be adapted to the endoscopic domain. In this work, we chose BYOL [4], but there are other similarly adequate methods such as EndoFM [5]. In Figure 5 we show how both methods are able to distribute similarly relevant scenes into different areas of the embedding space: for BYOL, Wall frames (upwards) and Surgery frames (downwards), and for EndoFM, NBI frames (downwards) and and Wall frames (leftwards). Qualitatively we conclude that the distribution of frames by EndoFM is conditioned by color more than with BYOL, since BYOL ignores if the image is captured with regular or NBI lighting. Additionally, the region with Surgery frames that is prominent in BYOL is of great interest for our applications. We also study the clustering possibilities for these embeddings. Table 2 contains the Silhouette Coefficient [6] and Davies-Bouldin Index [7] as quality metrics for the clusters obtained with different numbers of clusters ranging from 20 to 200. The values obtained do not change significantly with the number of clusters, so they are not useful for tuning said hyperparameter k. However, in both metrics we observe that the clustering using BYOL is more desirable that the one using EndoFM. Although EndoFM was trained as a foundation model for endoscopy images, the fine-tuning that we did with BYOL in EndoMapper proves to create more discriminative image descriptors in this dataset.

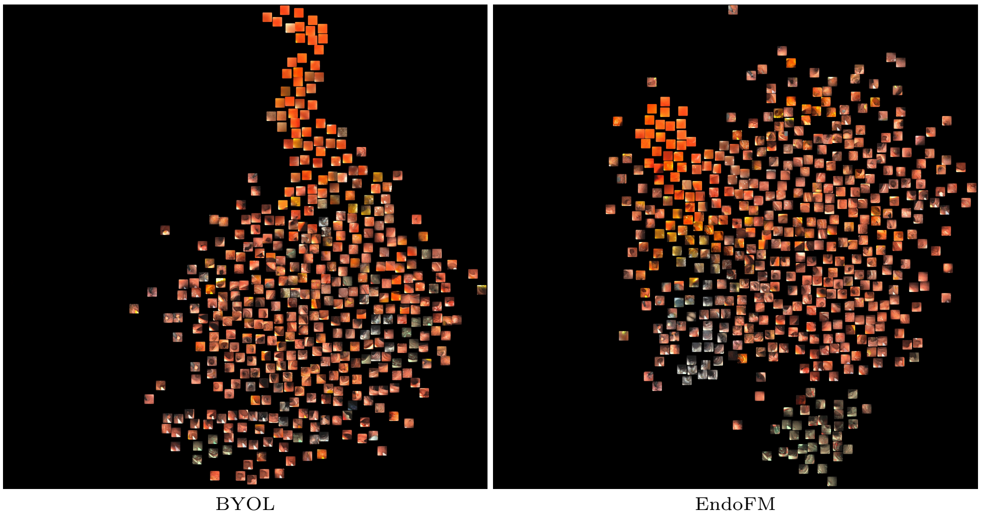

Fig. 5 t-SNE visualization of representative frames from **train-set-2**. The images are located using the descriptors obtained by two methods: BYOL (left) and EndoFM (right).

Table 2 Metrics for intra-cluster cohesion and inter-cluster distinction with two different image description methods (BYOL and EndoFM) and different number of clusters k.

**# Clusters (k)**	**20**	**50**	**75**	**100**	**150**	**200**
Silhouette Coefficient ↑	0.10	0.08	0.07	0.08	0.07	0.07
Davies-Bouldin Index ↓	2.25	2.30	2.30	2.29	2.28	2.27

(a) Clustering using BYOL [4]'s fine-tuned descriptors.

**# Clusters (k)**	**20**	**50**	**75**	**100**	**150**	**200**
Silhouette Coefficient ↑	0.05	0.04	0.04	0.04	0.04	0.05
Davies-Bouldin Index ↓	3.27	3.12	3.08	3.03	2.94	2.90

(b) Clustering using EndoFM [5]'s generic descriptors.

2.1 Qualitative error analysis: surgical action detection

Figure 6 contains frame samples of the only surgical action that our surgical action detection missed. This action has a duration of 39 seconds, where the surgical tool is only visible for the first few frames. The surgical action is visually characterized only by the polyp that is being resected, which is why our system is not able to detect the surgical tool that only appeared briefly. This signals that abnormality detection and disease detection could be symbiotic additions to our system.

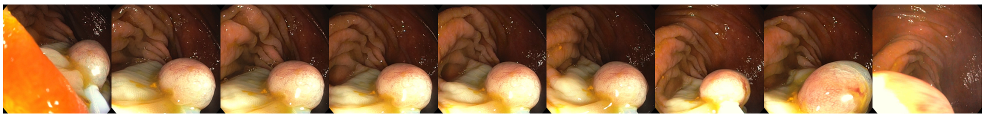

Fig. 6 Sample images of the only surgical action that our detection system missed. The frames are from test sequence **Seq_093** and are ordered from left to right in order of appearance.

2.2 Full video visual examples

Figures 8 and 9 contain the visibility conditions estimation for all sequences in **test-set**. Additional **gastroscopy** sequences are shown in Figure 7, outside of our method's domain since it was trained in colonoscopy.

References

[1] Azagra, P., Sostres, C., Ferrández, Á., Riazuelo, L., Tomasini, C., Barbed, O.L., Morlana, J., Recasens, D., Batlle, V.M., Gómez-Rodríguez, J.J., Elvira, R., López, J., Oriol, C., Civera, J., Tardós, J.D., Murillo, A.C., Lanas, Á., Montiel, J.M.M.: Endomapper dataset of complete calibrated endoscopy procedures. Scientific Data **10**(1), 671 (2023)

[2] Mei, Y., Yao, L., Jin, Q.: Ubiss: A unified framework for bimodal semantic summarization of videos. In: Proceedings of the 2024 International Conference on Multimedia Retrieval, pp. 1034–1042 (2024)

[3] Tomar, S.: Converting video formats with ffmpeg. Linux Journal **2006**(146), 10 (2006)

[4] Grill, J.-B., Strub, F., Altché, F., Tallec, C., Richemond, P., Buchatskaya, E., Doersch, C., Avila Pires, B., Guo, Z., Gheshlaghi Azar, M., Piot, B., Kavukcuoglu, K., Munos, R., Valko, M.: Bootstrap your own latent-a new approach to self-supervised learning. Advances in neural information processing systems **33**, 21271–21284 (2020)

[5] Wang, Z., Liu, C., Zhang, S., Dou, Q.: Foundation model for endoscopy video analysis via large-scale self-supervised pre-train. In: Int. Conf. on Medical Image Computing and Computer-Assisted Intervention, pp. 101–111 (2023). Springer

[6] Kaufman, L., Rousseeuw, P.J.: Finding Groups in Data: an Introduction to Cluster Analysis, p. 87. John Wiley & Sons, New York (2009)

[7] Davies, D.L., Bouldin, D.W.: A cluster separation measure. IEEE transactions on pattern analysis and machine intelligence (2), 224–227 (2009)

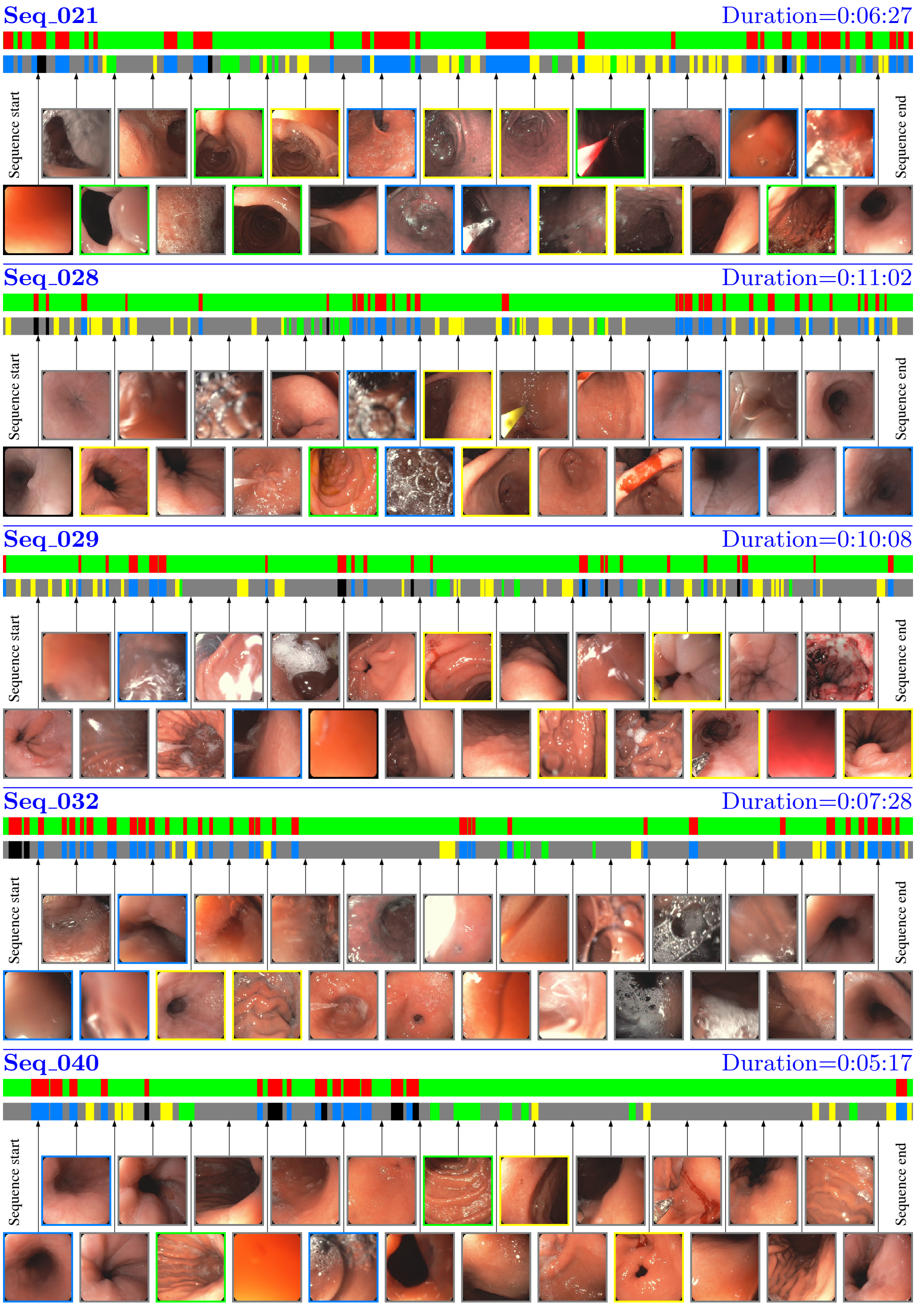

Fig. 7 Examples of automatic video overview obtained with our approach on a different domain: gastroscopy. Horizontal bars represent the video from start to end (left to right). The different colored sections are: Binary predictions for *Informative* (green) and *Non-Informative* (red). Fine grained predictions: *High quality* (green), *Medium quality* (yellow), *Liquids* (blue) and *Wall* (black). Bottom images are frame samples for different segments.

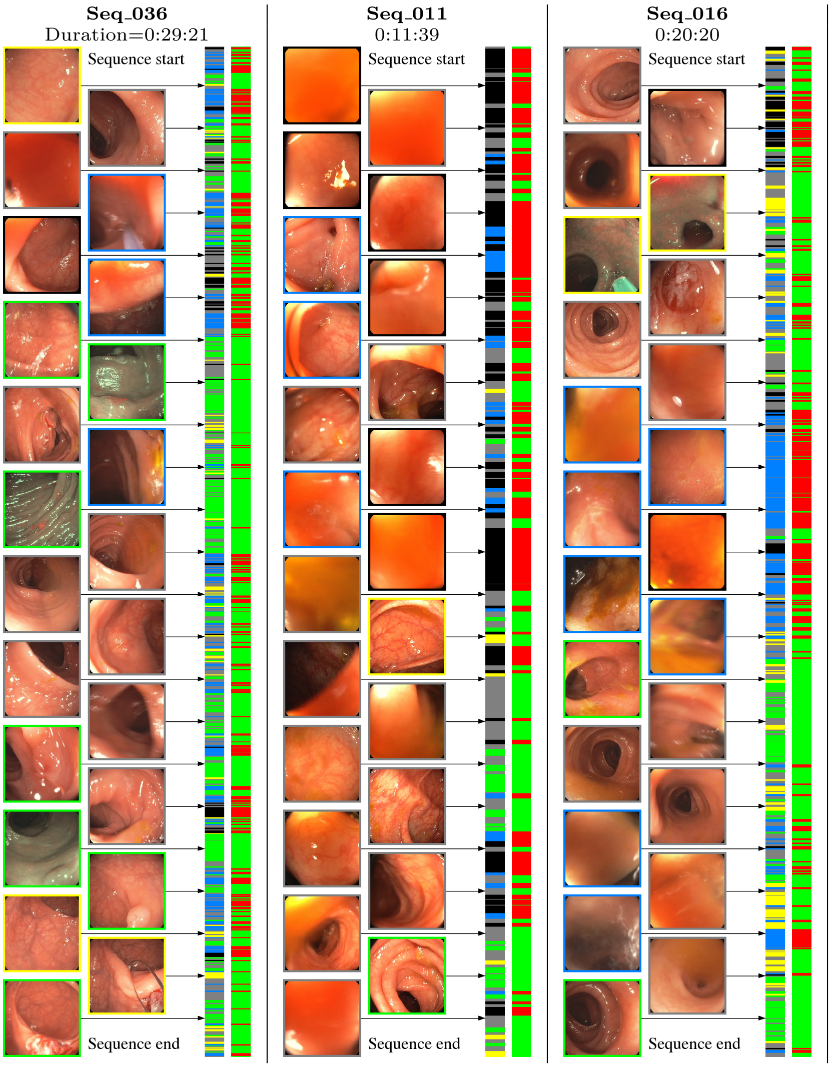

Fig. 8 Examples of visibility conditions estimation using our method. The colors in the first bar and the frame border represent the class of the segment: Green=*High quality*, Yellow=*Medium quality*, Blue=*Liquids*, Black=***Wall***, Gray=*Uncertain*. The colors in the second bar correspond to: Green=*Informative*, Red=*Non-Informative* (the *Uncertain* class is *Informative* for safety).

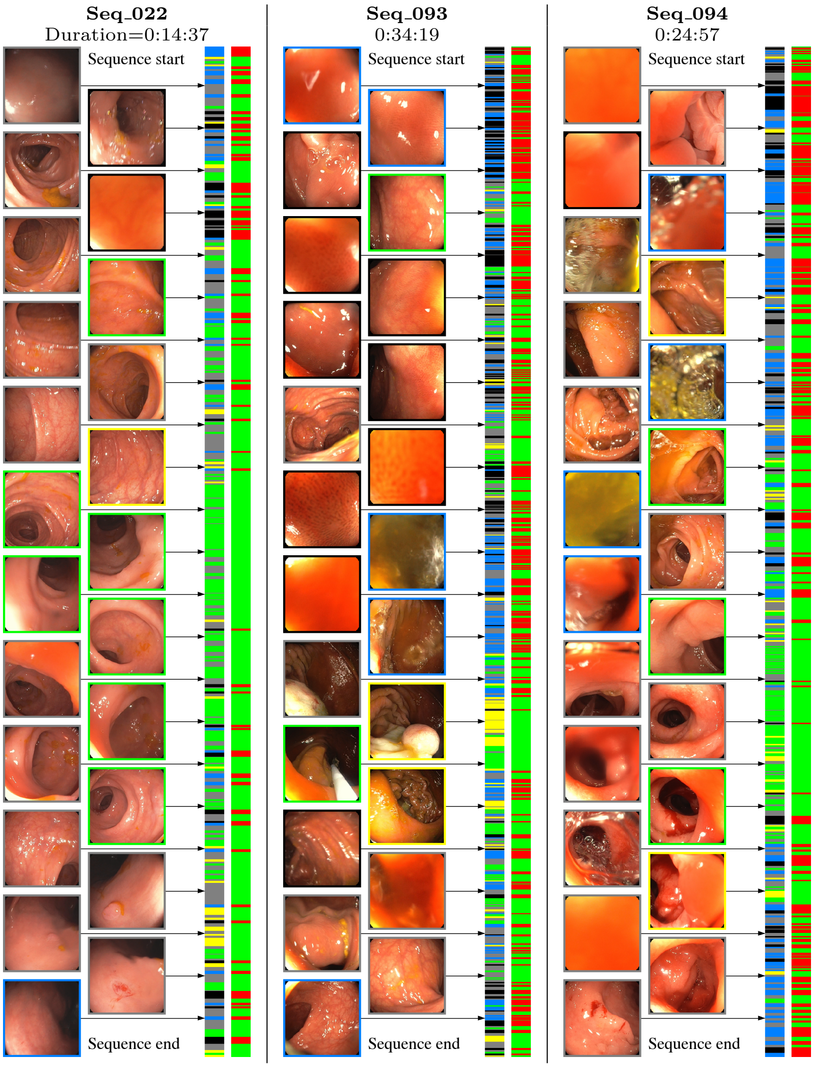

Fig. 9 Examples of visibility conditions estimation using our method. The colors in the first bar and the frame border represent the class of the segment: Green=*High quality*, Yellow=*Medium quality*, Blue=*Liquids*, Black=***Wall***, Gray=*Uncertain*. The colors in the second bar correspond to: Green=*Informative*, Red=*Non-Informative* (the *Uncertain* class is *Informative* for safety).
